# Supplementary material for: The interactome of intact mitochondria by cross-linking mass spectrometry provides evidence for coexisting respiratory supercomplexes
Source: Mol Cell Proteomics. 2017 Dec 8;17(2):216–32. doi: 10.1074/mcp.RA117.000470 (PMC5795388; doi:10.1074/mcp.RA117.000470)
Supplement: Supplemental Data [file supp_RA117.000470_133922_0_supp_23978_fzffwf.zip › spectra_annotation/mito_DR_spectra_annotation/331-1-3-1-2-1.pdf]

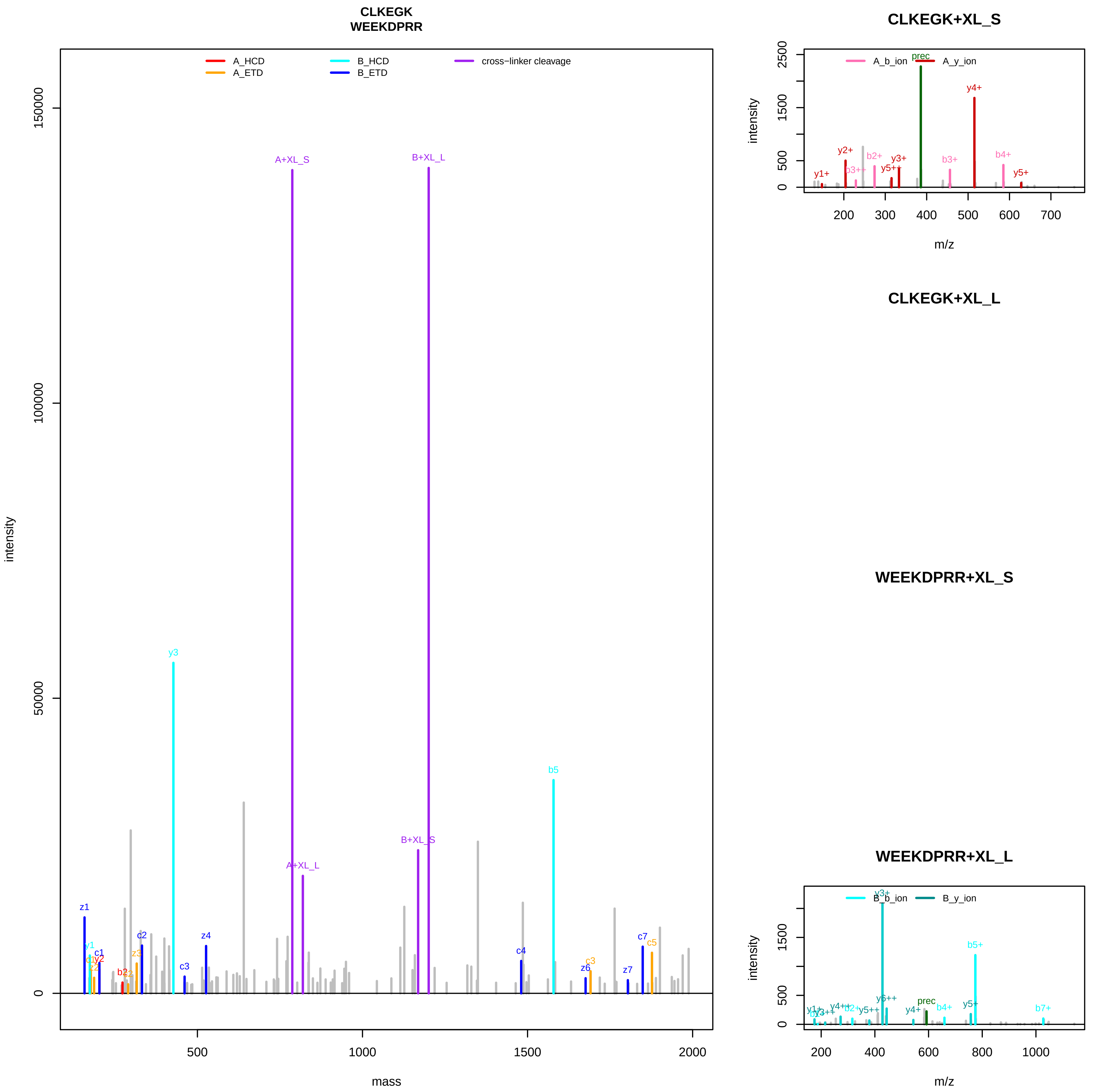
CLKEGK
WEEKDPRR
A_HCD
A_ETD
B_HCD
B_ETD
cross−linker cleavage
A+XL_S
B+XL_L
B+XL_S
A+XL_L
y3
b5
z1
c1
y2
b2
z2
z3
c2
c3
z4
c4
z6
c3
z7
c7
c5
intensity
mass
0
50000
100000
150000
500
1000
1500
2000
CLKEGK+XL_S
A_b_ion
A_y_ion
prec
y4+
y2+
b2+
y3+
b3++
y5++
b3+
b4+
y1+
y5+
intensity
m/z
0
500
1500
2500
200
300
400
500
600
700
CLKEGK+XL_L
WEEKDPRR+XL_S
WEEKDPRR+XL_L
B_b_ion
B_y_ion
y3+
b5+
y6++
prec
y4++
b2+
y5++
y4+
b4+
y5+
b7+
y1+
y3++
intensity
m/z
0
500
1500
200
400
600
800
1000
